# The Calcium-Sensing Receptor and the Reproductive System

**DOI:** 10.3389/fphys.2016.00371

**Published:** 2016-08-30

**Authors:** Isabella Ellinger

**Affiliations:** Pathophysiology of the Placenta, Department of Pathophysiology and Allergy Research, Center for Pathophysiology, Infectiology and Immunology, Medical University ViennaVienna, Austria

**Keywords:** calcium-sensing receptor, reproduction, testes, ovaries, uterus, placenta

## Abstract

Active placental transport of maternal serum calcium (Ca^2+^) to the offspring is pivotal for proper development of the fetal skeleton as well as various organ systems. Moreover, extracellular Ca^2+^ levels impact on distinct processes in mammalian reproduction. The calcium-sensing receptor (CaSR) translates changes in extracellular Ca^2+^-concentrations into cellular reactions. This review summarizes current knowledge on the expression of CaSR and its putative functions in reproductive organs. CaSR was detected in placental cells mediating materno-fetal Ca^2+^-transport such as the murine intraplacental yolk sac (IPYS) and the human syncytiotrophoblast. As shown in *casr* knock-out mice, ablation of CaSR downregulates transplacental Ca^2+^-transport. Receptor expression was reported in human and rat ovarian surface epithelial (ROSE) cells, where CaSR activation stimulates cell proliferation. In follicles of various species a role of CaSR activation in oocyte maturation was suggested. Based on studies in avian follicles, the activation of CaSR expressed in granulosa cells may support the survival of follicles after their selection. CaSR in rat and equine sperms was functionally linked to sperm motility and sperm capacitation. Implantation involves complex interactions between the blastocyst and the uterine epithelium. During early pregnancy, CaSR expression at the implantation site as well as in decidual cells indicates that CaSR is important for blastocyst implantation and decidualization in the rat uterus. Localization of CaSR in human extravillous cytotrophoblasts suggests a role of CaSR in placentation. Overall, evidence for functional involvement of CaSR in physiologic mammalian reproductive processes exists. Moreover, several studies reported altered expression of CaSR in cells of reproductive tissues under pathologic conditions. However, in many tissues we still lack knowledge on physiological ligands activating CaSR, CaSR-linked G-proteins, activated intracellular signaling pathway, and functional relevance of CaSR activation. Clearly, more work is required in the future to decode the complex physiologic and pathophysiologic relationship of CaSR and the mammalian reproductive system.

## Introduction

Calcium (Ca^2+^) is indispensable in the context of mammalian reproduction (Baczyk et al., 2011; Correia et al., 2015; Kornbluth and Fissore, 2015). Firstly, Ca^2+^ contributes to crucial developmental processes such as skeletal formation and mineralization (Riccardi et al., 2013; Kovacs, 2014, 2015), lung (Riccardi et al., 2013) and kidney development (Gilbert et al., 2011) or formation and maturation of neuronal circuits and long-term memory (Leclerc et al., 2011). Therefore, Ca^2+^ must be supplied in sufficient quantities to the growing offspring, which is accomplished by active Ca^2+^-transport from maternal to fetal or neonate blood circulation across placental and mammary tissue, respectively (Olausson et al., 2012; Kovacs, 2014, 2015, 2016). Secondly, Ca^2+^ is the most universal second messenger. It is modulated downstream of numerous receptors and can activate diverse cytoplasmic signaling proteins (Berridge et al., 2000). Not surprisingly, therefore, Ca^2+^ signaling pathways also play crucial roles in early reproductive events like gamete formation and maturation in the male and female gonads, and fertilization as well as pre- and peri-implantation development in the female reproductive tract (Kashir et al., 2013; Armant, 2015).

Both, extracellular and intracellular Ca^2+^-linked processes require a tightly regulated extracellular calcium (Ca${}_{\text{o}}^{2+}$) concentration. Mammals have therefore developed a carefully balanced Ca${}_{\text{o}}^{2+}$ homeostatic system, which is based on Ca${}_{\text{o}}^{2+}$-sensors. The calcium-sensing receptor (CaSR), is the master regulator of Ca${}_{\text{o}}^{2+}$ concentration (Brown, 2013; Tyler Miller, 2013; Alfadda et al., 2014). CaSR controls secretion of a regulatory hormone, parathyroid hormone (PTH), that in turn impacts on Ca${}_{\text{o}}^{2+}$ via cells in the target tissues kidney, intestine, and bone (Brown, 2013).

CaSR is additionally expressed in other adult tissues, including the central and peripheral nervous system (Ruat and Traiffort, 2013; Jones and Smith, 2016), the cardio-vascular system (Smajilovic et al., 2011; Schepelmann et al., 2016), the lung (Riccardi et al., 2013), the pancreas (Squires et al., 2014), the epidermis (Tu and Bikle, 2013), or the intestine (Macleod, 2013). There, the function of CaSR is not related to control of Ca${}_{\text{o}}^{2+}$ -homeostasis. Instead, CaSR modulates functions such as proliferation and differentiation, apoptosis and chemotaxis, ion channel activity, or hormone secretion, to name a few.

The outstanding role of Ca^2+^ in reproduction together with CaSR expression in reproductive organs implicates a role of CaSR in reproductive processes. This review first introduces CaSR and its functional versatility. It then gives a survey on organs and processes required for reproduction, and summarizes the still sparse information on expression, localization, and function of CaSR in gametes, gonads, uterus, and placenta in health and disease (summarized in Table 1). Finally, it indicates research demand in these areas. Expression and function of CaSR in mammary epithelial cells is not addressed in this article as this has been reviewed recently (Kovacs, 2016). Likewise, the role of CaSR in proper development of the skeleton (Riccardi et al., 2013; Kovacs, 2014), the lung (Riccardi et al., 2013; Brennan et al., 2016) and the brain (Liu et al., 2013) is not considered in this article.

**Table 1:** **CaSR expression and putative functions in healthy reproductive tissues**.

**Organ/Tissue**	**Species**	**mRNA**	**Anti-CaSR antibody**	**Protein WB**	**Protein IFM/IHC**	**Function related to CaSR**	**References**
**MALE REPRODUCTIVE SYSTEM**							
	Rat	Testis Epididymis	Rabbit anti-CaSR Acris (N-terminal domain)		Spermatogonia Spermatocytes Spermatids Sertoli cells Epididymal cells	Sperm motility ↑	Mendoza et al., 2012
	Horse		Goat anti CaSR (F19) Santa Cruz (N-terminal domain)	Sperm 100 kDa + 77 kDa	Sperms	Sperm capacitation ↓ Sperm motility ↑	Macías-García et al., 2016
	Human		Rabbit anti-CaSR Sigma (N-terminal domain)		Epithelial cells in normal prostate tissue		Feng et al., 2014
**OVAR**							
	Human	Ovarian surface epithelial cell lines	Polyclonal anti-CaSR Affinity BioReagents		Ovarian surface epithelial cell lines,120 kDa	Pro-liferation ↑ $Ca_{\text{I}}^{2+}$↑ IP3 ↑	McNeil et al., 1998
	Human		Rabit anti-CaSR (C0117-15) US Biological (C-terminal)	Denuded oocyte (metaphase II),130 kDa Granulosa cells of cumuli oophori cells, 120 + 130 kDa	Oocyte (GV, MI, MII state)		Dell'Aquila et al., 2006
	Horse		Rabit anti-CaSR (C0117-15) US Biological (C-terminal)	Denuded oocyte (metaphase II), 130 kDa Granulosa cells of cumuli oophori cells, 120 + 130 kDa	Oocyte (metaphase II) Granulosa cells of cumuli oophori cells	Oocyte maturation↑	De Santis et al., 2009
	Pig	Ocytes Granulosa cells of Cumuli oophori	Goat anti-CaSR Santa Cruz	Oocyte, 160 kDa Somatic cell, 160 kDa		Oocyte maturation↑	Liu et al., 2015
	Japanese Quail		Mouse anti-CaSR Abcam or NPS Pharmaceutical (Extracellular domain)	Granulosa explants 115–125 kDa +100–110 kDa	Granulosa cells of preovulatory follicles Granulosa layer after ovulation	Survival↑ (Decreased apoptosis)	Diez-Fraile et al., 2010
**UTERUS**							
	Rat	Uterine luminal epithelium Uterine stromal cells	Rabbit anti-CaSR Affinity BioReagents		Implanting blastocyst Luminal and glandular epithelium Subluminal uterine stromal cells at implantation site	Blastocyst implantation Decidua-lization	Xiao et al., 2005
	Rat		Anti-CaSR Affinity BioReagents		Uterine luminal Epithelium Longitudinal and circular smooth muscle layers Blood vessels Luminal and glandular epithelium of the endometrium	Relaxation?	Pistilli et al., 2012
	Human		Anti-CaSR (ab19347) Abcam		Uterine Myometrium (low) Human term placenta	Relaxation?	Crankshaw et al., 2013
**PLACENTA**							
	Mouse	Intra-placental yolk sac cells, parietal side + columnar side	Mouse anti-CaSR ADD		Intraplacental yolk sac cells, parietal side + columnar side Placental trophoblasts		Kovacs et al., 2002
	Human		Anti-CaSR Novus HL-1499 (WB) anti-CaSR Acris (IHC)	Term Placenta 130 kDa	Term villous STB Term villous cytotrophoblast Term extravillous trophoblast		Papadopoulou et al., 2014
	Human	First trimester villous STB	Monoclonal Anti-CaSR NPS Pharmaceuticals, human extracellular CaSR epitopes amino acids 214–235 or amino acids 374–391		Villous STB (first trimester and term) Extravillous trophoblast (first trimester and term)	$Ca_{\text{I}}^{2+}$↑	Bradbury et al., 2002
	Human	Extravillous cytotrophoblast					Bradbury et al., 1998

## The calcium sensing receptor, CaSR, and its functional versatility

CaSR is a member of the class C of the G protein-coupled receptors and is present in all vertebrate classes. The fully glycosylated monomer has a molecular mass of 160 kDa and consists of a large N-terminal glycosylated extracellular domain, a seven transmembrane domain and an intracellular C-terminal domain. The extracellular domain contains a bi-lobed Venus-flytrap-like domain. The functional receptor is a dimer, where the Venus-flytrap-like domains of two monomers are linked via covalent as well as non-covalent interactions. Ca^2+^-binding in the cleft between the two lobes of each Venus-flytrap-like domain causes a rotation of one monomer relative to the other, which finally allows for G proteins to interact with the cytoplasmic side of the CaSR (Zhang et al., 2015).

CaSR is, however, promiscuous in ligand binding; it can be stimulated by other divalent (Mg^2+^) and tervalent (Gd^3+^) inorganic cations, as well as organic polycations (e.g., neomycin, spermine). Moreover, it is modulated by various physiological stimuli including extracellular pH, L-aromatic amino acids, and ionic strength. Different ligand binding sites can stabilize distinct conformational changes, which results in “ligand-biased signaling.” As a consequence, CaSR was shown to interact with the heterotrimeric G proteins G_q/11_, G_i/o_, G_12/13_, and G_s_ and is able to target all major intracellular signaling pathways. CaSR-mediated activation of G_q/11_ results in stimulation of phospholipase C (PLC), Ca${}_{\text{i}}^{2+}$ mobilization from intracellular stores, and activation of protein kinase C (PKC) isoforms. CaSR-coupling to G_i/o_ can inhibit adenylyl cyclase (AC). On the other hand, it can activate mitogen-activated protein kinases (MAPK) such as ERK1/2 and JNK. This can lead to transactivation of the epidermal growth factor receptor (EGFR). Activation of G_12/13_ modulates several pathways. This can lead to migration via rho-mediated actin polymerization and membrane ruffling or induce cell differentiation. It can also target tyrosine kinases, protein phosphatases, or activate certain AC isoforms. CaSR-coupling to G_s_ also activates ACs. Furthermore, CaSR activation can stimulate PLA, phosphatidylinositol 3-kinase (PI-3K) and PI-4K. Overall, major consequences of CaSR activation in cells are Ca${}_{\text{i}}^{2+}$ mobilization, regulation of intracellular cAMP levels, activation of various protein kinases as well as activation of gene transcription factors. CaSR-mediated signaling, however, depends on the cell-type-specific expression of important components of the downstream signaling pathways (Conigrave and Ward, 2013).

An example for the cell-type specific function of CaSR is the contradictory role in cancer development, where it acts as either an oncogene (breast, prostate) or as a tumor suppressor gene (colon, parathyroid) (Brennan et al., 2013; Peterlik et al., 2013; Tennakoon et al., 2016). CaSR activation can also have diametrical consequences in normal and tumor cells as shown for mammary epithelial cells by activating different G-proteins (Mamillapalli et al., 2008). The multiple consequences of CaSR activation are illustrated by its variable impact on intracellular Ca^2+^ concentration. CaSR can directly increase cytosolic Ca^2+^ through PLC activation to open Ca^2+^ channels. Alternatively, CaSR can trigger ERK/phospho-ERK pathways, thereby increasing transcription factor (e.g., CREB) activity, which stimulates expression of Ca^2+^-channels with appropriate response elements. And finally, CaSR can increase K^+^-channel activity, and as a consequence increase membrane potential and a Ca^2+^-driving force. Many intracellular signaling pathways, in turn, are sensitive to cytosolic Ca^2+^-rises, and as a consequence, cellular processes such as proliferation or migration can result from CaSR activation (Tennakoon et al., 2016).

Overall, CaSR has immense functional versatility by activating different signaling pathways in a ligand- and cell-type specific way. In the context of reproduction (see below), mainly activation of MAPK pathways (e.g., ERK1/2, p38, or JNK) has been investigated so far, probably because MAPK activation is of great importance in reproduction (Li et al., 2009; Almog and Naor, 2010; Fan et al., 2012; Nunes et al., 2015). However, CaSR can also induce other pathways such as the PI-3K/AKT pathway (Bilderback et al., 2002; Liao et al., 2006) in the reproductive system. Activation of diverse intracellular signaling routes should be considered in future studies addressing the function of CaSR in reproduction.

## CaSR and the male reproductive system

### Spermatogenesis

The mass production of the male gamets starts with puberty, when the hypothalamic-pituitary-gonadal axis is established, and is a continuous, life-long process. Spermatogenesis consists of three major phases (1) the proliferation of the stem cells (spermatogonia) resulting in spermatocytes, (2) two meiotic divisions that give rise to haploid spermatids, and (3) spermiogenesis, the differentiation into mature sperms (for details see Kornbluth and Fissore, 2015). Spermatogenesis occurs in the seminiferous tubulus of the testes and is supported by testicular non-germ line cells, such as Sertoli and Leydig cells, which nourish the sperms and/or produce factors required for proper spermatogenesis. Within the epididymis the sperms complete maturation. Secretory products of the male accessory sex glands (e.g., prostate gland) support the functionality of sperms. Once in the female tract, sperms undergo capacitation to increase motility (hyperactivation) and prepare for fertilization of the oocyte. Only capacitated sperms can bind to molecules in the egg's zona pellucida, which triggers the acrosome reaction. Enzymes released from the acrosome, a Golgi-derived sperm organelle, digest a way through the zona pellucida enabling the sperm to follow and to finally fertilize the egg (de Kretser, 2012). Ca^2+^ is a major determinant of proliferation and differentiation of spermatogonia, sperm motility, capacitation, and acrosome reaction (Breitbart, 2002; Yoshida and Yoshida, 2011; Correia et al., 2015; Kornbluth and Fissore, 2015). In contrast to Ca^2+^ channels, which are well studied in sperms (Correia et al., 2015), sparse information is available on the expression and function of CaSR in sperm or male reproductive tissues (see also Table 1).

### CaSR expression in healthy male reproductive tissue

**Figure 1:**
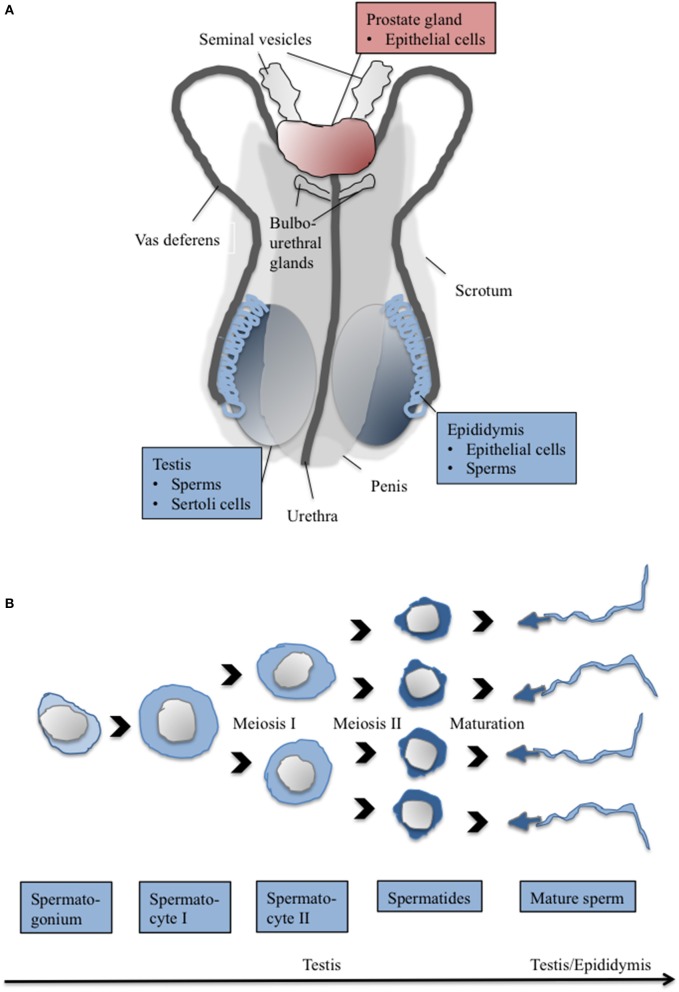
**The cartoon depicts (A) major components of the human male reproductive system and (B) major steps in spermatogenesis**. Organs/tissues/cells, which were shown to express CaSR are depicted in color. Red color, CaSR expression shown in human ± other mammalian species; blue color, CaSR expression shown in mammalian species other than human (for details on species and assumed function of CaSR see Text and Table 1).

In rats, CaSR mRNA expression appeared higher in testes compared to epididymis (Figure 1A). In testis, CaSR protein was found in Sertoli cells, spermatogonia, spermatocytes, and spermatids with highest expression level in spermatids (Figure 1B). Leydig cells and fibroblasts, in contrast, presented CaSR negative. Mature sperms in the epididymis also expressed CaSR, predominantly at the head domain. Epithelial epididymal cells exhibited staining at the apical pole. Since a calcimimetic drug (AMG 641) caused a moderate, but significant increase of motility of isolated rat sperms, a role of CaSR in sperm motility was suggested. A similar stimulatory effect on pig sperms mobility indicated expression of CaSR also in male gametes of this species (Mendoza et al., 2012).

In isolated equine sperms, CaSR protein was located predominantly in the head of the sperm, with lower expression seen along the tail. Proteins detected with an anti-CaSR antibody by western blotting had a molecular weight of 100 and 75 kDa (Macías-García et al., 2016), which is smaller than that of fully glycosylated CaSR transiently expressed in HEK cells (160 kDa) (Pidasheva et al., 2006). The authors suggested involvement of CaSR in sperm capacitation as well as sperm mobility. Capacitation occurs when alterations in the extracellular milieu induce cAMP and activate PKA. The result is tyrosine phosphorylation (PY) of various proteins. Among factors impacting on PY is Ca${}_{\text{o}}^{2+}$. In species such as stallion (González-Fernández et al., 2013), human and mice (Baker et al., 2004), Ca${}_{\text{o}}^{2+}$ in the capacitation medium inhibits PY. Ca^2+^-dependent PY inhibition in stallion sperm was reversed in the presence of a CaSR antagonist (NPS 2143), while a CaSR agonist (AC-265347) inhibited PY similarly to Ca${}_{\text{o}}^{2+}$. The result suggested that CaSR reduces capacitation in stallion sperm. A modulatory role of the pH was observed. In addition, NPS 2143 caused a significant decrease in sperm motility indicating the CaSR is also involved in the regulation of equine sperm mobility (Macías-García et al., 2016).

### CaSR and disorders of the male reproductive system

Diabetes, being an increasing problem worldwide^1^, can cause testicular damage and male infertility. A recent study demonstrated up-regulated CaSR in testicular tissues in streptozotoxin-induced diabetic rats (Kong et al., 2016). The diabetic rats had significantly lower testes weights and serum levels of testosterone compared to healthy controls. A connection between CaSR activation and testicular damage was found. Specific activation of CaSR by Gd^3+^ increased, while specific inhibition of CaSR by the antagonist NPS 2390 reduced testicular damage in the diabetic rats. Increased lipid peroxidation, decreased anti-oxidative capacity, increased apoptosis of germ cells, and activation of the mitochondrial apoptotic pathways were observed in testicular tissue of diabetic rats and the parameters were further aggravated by the administration of Gd^3+^, and attenuated by NPS 2390. CaSR was found to be an activator of different MAPK pathways (ERK, p38, JNK). It was concluded that CaSR activation had a pro-apoptotic impact on germ cells in diabetic rats and overall participated in diabetes-induced testicular damage.

While CaSR expression in normal rat Leydig cells could not be shown (Mendoza et al., 2012), cultured Rice H-500 rat Leydig cell tumor express the receptor mRNA and protein (Sanders et al., 2000). H-500 cells are a transplantable model for humoral hypercalcemia of malignancy. Humoral hypercalcemia of malignancy is a syndrome seen in various types of cancers including prostate cancer, but also testicle or breast cancer. It arises from tumor-derived humoral factors which destroy normal calcium homeostasis (Chakravarti et al., 2009). Upon implantation into adult male rats, H-500 cells start proliferation and cause hypercalcemia by abundant release of the humoral factor parathyroid hormone-related protein (PTHrP). PTHrP release, proliferation, and protection of cells from apoptosis, are stimulated by Ca${}_{\text{o}}^{2+}$ via CaSR. CaSR-induced proliferation depends on AKT and MAPK p38, but not on MAPK ERK1/2 (Tfelt-Hansen et al., 2004). CaSR also stimulates transcription of the PTHrP gene, and release of PTHrP in H-500 cells by activating PKC as well as various MAPK pathways (ERK1/2, p38, JNK) (Tfelt-Hansen et al., 2003a). Partly, these effects are caused by transactivation of EGFR by CaSR (Tfelt-Hansen et al., 2005). In addition to PTHrP, Ca${}_{\text{o}}^{2+}$ also up-regulates mRNA of the oncogene pituitary tumor transforming gene as well as vascular endothelial growth factor genes in H-500 cells, which leads to the robust proliferation and angiogenesis at the site of their implantation (Tfelt-Hansen et al., 2003b). Stimulation of PTHrP release initiates a vicious circle of hypercalcemia maintained by CaSR expression in Leydig cell tumors, which suggests CaSR as a potential therapeutic target for CaSR antagonists. Colloton and coworkers investigated the ability of cinacalcet, an allosteric modulator of CaSR, to attenuate hypercalcemia in mice bearing H-500 cells and indeed demonstrated that cinacalcet effectively reduced tumor-mediated hypercalcemia (Colloton et al., 2013).

In an immunohistochemical study, comparing human prostate cancer tissue sections in microarrays, CaSR expression was confirmed not only in primary prostate cancer tissue, but also in normal prostate tissue (Figure 1A). No significant difference in the CaSR expression level between these tissues was observed. However, a significant higher expression level of CaSR was found in the metastatic prostate cancer tissues obtained from bone (Feng et al., 2014). For breast cancer cells it was demonstrated that the elevated Ca^2+^-release during bone remodeling represents a chemoattractant, which promotes migration of cancer cells to bone via activated CaSR (Saidak et al., 2009). CaSR mRNA and protein is also detected in the highly bone metastatic prostate cancer cell lines PC-3 and C4-2B, while comparatively lower expression of CaSR is found in non-skeletal metastatic, epithelial-derived prostate cell line LNCaP cells (Liao et al., 2006). Prostate cancer most commonly metastasizes to bone, preferring areas of high bone turnover. As a result of bone remodeling, Ca^2+^ and growth factors are released. In PC-3 and C4-2B, but not LNCaP cells elevated Ca${}_{\text{o}}^{2+}$ activated CaSR. Activation resulted in stabilization of cyclin D, promoted PI-3K/AKT/ mTOR signaling, and increased metastatic potential (Liao et al., 2006). A few clinical studies supported the notion that CaSR promotes lethal prostate cancer. First, the CaSR Q1011E minor allele, which is common in populations with African ancestry, appeared to be associated with a less aggressive form of prostate cancer among African-American men (Schwartz et al., 2010). A second study assessed genetic variations across CaSR and lethal prostate cancer risk in Caucasian men. Common genetic variations in CaSR were found associated with both higher and lower risk for lethal prostate cancer. The association was stronger in patients with lower plasma levels of vitamin D (Shui et al., 2013). Third, in a study that correlated primary tumor CaSR expression with the risk for lethal prostate cancer, a higher CaSR tumor expression was associated with an approximately two-fold higher risk for lethal progression. This risk was independent of Gleason grade and pathological stage. Higher CaSR expression was significantly associated with lethal progression among cases with lower tumor vitamin D receptor expression but not among cases with high tumor vitamin D receptor expression (Ahearn et al., 2016).

### Research demands

Reports on CaSR expression in human male reproductive system are currently mainly related to cancer. For more detailed information on the role of CaSR in tumors, the reader is referred to other reviews (Singh et al., 2013; Mateo-Lozano et al., 2016; Tennakoon et al., 2016). The high CaSR expression in Leydig and prostate cell tumors asks for further evaluation of the prognostic usability of CaSR. As CaSR promotes bony metastasis of cancer, this raises the possibility of reducing the risk of such metastases with CaSR-based therapeutics.

Infertility is among the most serious social problems affecting advanced nations today. Infertility affects both men and women. A variety of known factors is associated with male infertility, but in 30–45%, the cause of the abnormal semen parameters is not identified (Jungwirth et al., 2012). In this context, knowledge of all molecules that impact on proper sperm development is of relevance. If CaSR would be demonstrated to significantly improve or reduce sperm quantity or quality, agonists or antagonists of the receptor, respectively, could serve for treatment. But currently, many open questions remain. The intracellular signaling pathways associated with the observed effect of CaSR on (rat, porcine, and equine) sperm motility and capacitation remain to be investigated. The predominant physiological ligands responsible for these effects are unknown. Of interest, Ca^2+^ concentrations in the uterine fluid vary during estrous cycle in various species (Casslén and Nilsson, 1984; Alavi-Shoushtari et al., 2012; Alavi Shoushtari et al., 2014). Polyamines (spermine, spermidine, and putrescine), which are type I calcimimetics (Brown, 2010) are secreted by the prostate gland into semen. Whether polyamines, or other CaSR ligands in the semen could be sensed by CaSR expressed on sperms and have effects, is not known. It is unknown, whether CaSR under physiological conditions impacts on sperm cell apoptosis. Likewise, CaSR may modulate acrosome reaction or spermatic hyperactivation. The function of CaSR expressed in (rat) Sertoli cells and epididymal cells remains to be investigated. CaSR expression was not observed in healthy rat Leydig cells. However, there is functional evidence for a divalent cation (Ca^2+^) receptor present on the surface of murine cells inducing Ca^2+^ release from ryanodine receptor-gated intracellular Ca^2+^ stores (Adebanjo et al., 1998). Since a raise of extracellular Ca^2+^ can induce testosterone secretion of Leydig cells (Meikle et al., 1991), re-evaluation of CaSR expression in Leydig cells in mammalian species might be of interest when looking for new therapies to increase or reduce hormone secretion. The molecular size of CaSR protein detected in equine sperm is small compared to the mature glycosylated CaSR. The reason for this difference is currently unknown. It may relate to different glycosylation of CaSR or alternatively spliced CaSR in the tissue. In addition, a recent study indicated that the western blotting profiles in tissues depend on the anti-CaSR antibody used (Graca et al., 2016). Various anti-CaSR antibodies were used in the studies summarized in this review. They are listed in Table 1.

## CaSR in ovary and oocytes

### Ovaries and oogenesis

Only a small number of mature oocytes is released during the reproductive years of a woman. Ovulation occurs in cycles consisting of follicular phase, ovulation and luteal phase. The follicular phase is characterized by estrogen dominance, while in the luteal phase, progesterone is the dominant sex hormone. Due to the cyclic release of the steroids, the female body exhibits cyclicity, which is accompanied by structural and functional changes in the ovaries, oviducts, and the uterus. In most animals, this is called estrous cycle, while higher primates including human experience menstrual cycles. The reason of the changes occurring during female cyclicity is the dual function of the female reproductive tract. Under estrogen dominance, the body is prepared to receive the male gametes and enable fertilization, while under progesterone dominance the body is prepared for implantation and nourishment of the conceptus (for details see Findlay, 2012).

The ovarian surface is covered with a single layer of epithelial cells. Ovarian surface epithelial cells are responsible for the most malignant forms of ovarian carcinoma. In the ovarian cortex, ovarian follicles of various sizes and at different stages of development are located. The follicles are the functional units of the ovary. They contain a single oocyte and are surrounded by epithelial granulosa cells. Theca cells are closely associated with the follicles. Theca cells differentiate from the interfollicular stroma due to signals from growing follicles and produce the androgen substrate, which is required for estrogen biosynthesis in the granulosa cells. After ovulation, theca cells are transformed from androgen-producing to progesterone-producing cells, thereby becoming the pregnancy-maintaining cells of the corpus luteum. The polycystic ovary syndrome (PCOS) is characterized by hyperandrogenism, oligo- and/or anovulation, and polycystic ovarian morphology. PCOS is associated with insulin resistance, hyperinsulinemia and central obesity. Excessive proliferation of theca cells is often associated with PCOS; this is a major cause of infertility due to ovarian hyperandrogenism (Magoffin, 2005).

**Figure 2:**
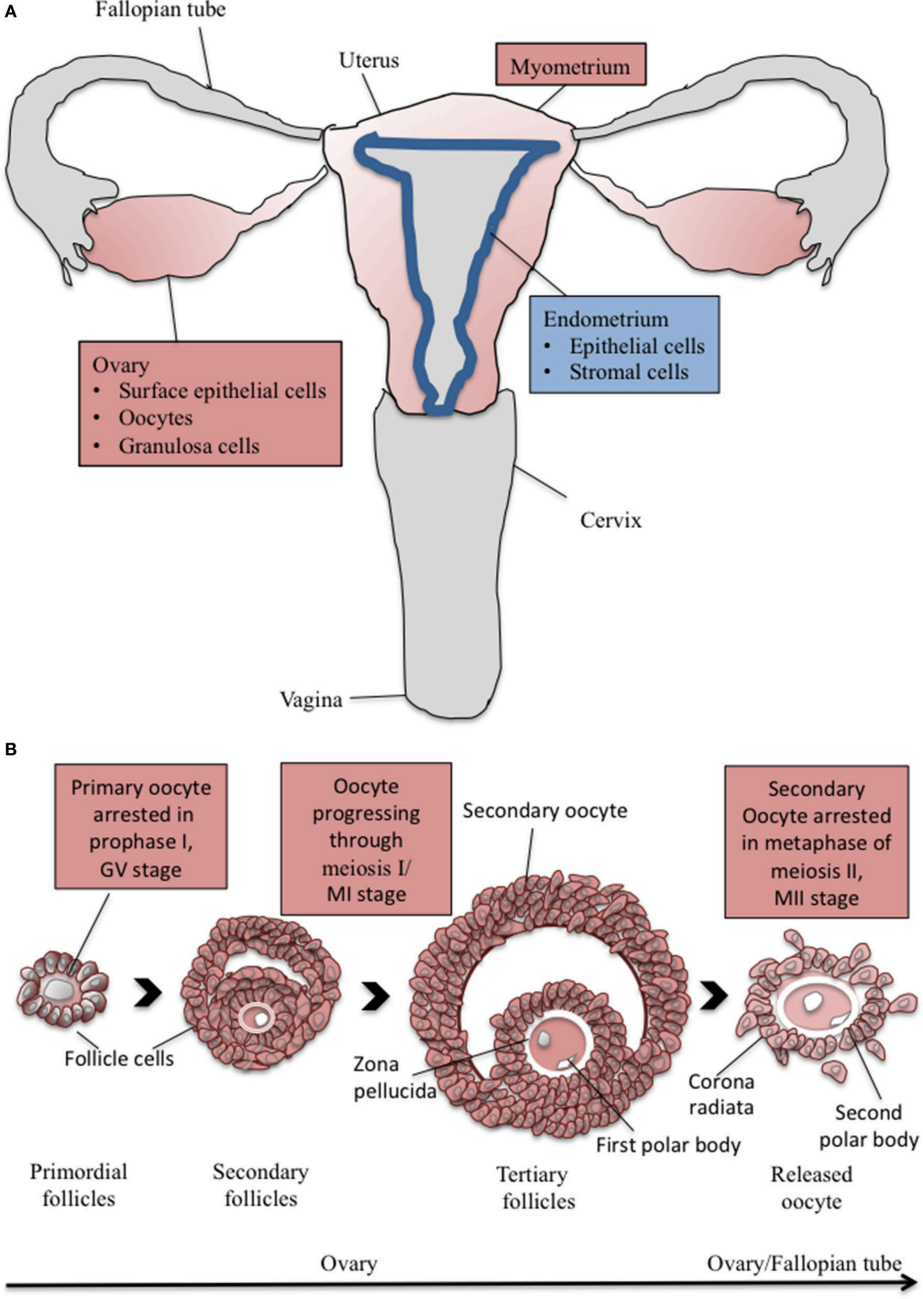
**The cartoon depicts (A) major components of the human female reproductive system and (B) major steps in oogenesis**. Organs/tissues/cells, which were shown to express CaSR are depicted in color. Red color, CaSR expression shown in human ± other mammalian species; blue color, CaSR expression shown in mammalian species other than human (for details on species and assumed function of CaSR see Text and Table 1).

Oogenesis starts with the formation of primary oocytes during the fetal period *in utero*. Stem cells (oogonia) first proliferate and then enter the first meiotic division, which is halted in a prophase state of meiosis I until sexual maturity. This first meiotic arrest is characterized by a large nucleus called the germinal vesicle (GV see Figure 2B). The oocyte and the surrounding granulosa cells, together constituing the primordial follicles, have complex paracrine interactions during follicle growth and development. Oocyte maturation depends on secretory products of the surrounding cells (Findlay, 2012).

Oocyte maturation—defined as the period of progression from the first to the second meiotic arrest—starts with puberty, when during the follicular phase of each menstrual/estrous cycle, a pool of follicles is recruited. Hormonal signals stimulate the oocytes as well as their surrounding granulosa cells to grow and theca cells attach to the follicle. One dominant follicle continues growing. Increasing estrogen levels produced by the follicle's granulosa cells cause a pulsatile release of luteinizing hormone (LH) from the pituitary gland, which stimulates the release of the oocyte from the ovary (ovulation). Upon that stimulus, meiosis I continues and is terminated shortly before ovulation. Oocytes at metaphase I (MI) stage are defined as oocytes with no GV (intact nucleus) and no polar body (see Figure 2B). Granulosa cells and the oocyte cooperate and transmit signals involved in maintaining or releasing the meiotic arrest in the oocyte. The arrest in prophase I is mediated by elevated levels of cyclic adenosine monophosphate (cAMP). Activation of MAPKs triggers degradation of cAMP, allowing oocyte maturation to proceed. Ca^2+^ plays a role in controlling LH-induced as well as spontaneous oocyte maturation, possibly by modulating intracytoplasmic cAMP concentrations via Ca^2+^-sensitive AC (Silvestre et al., 2011, 2012; Chen et al., 2013). By termination of meiosis I, the diploid primary oocyte generates two haploid daughter cells. One cell becomes the secondary oocyte (MII stage of oocyte), while the other cell forms the first polar body (see Figure 2B). The released oocyte is surrounded by the zona pellucida, a thick specialized extracellular matrix as well as additional layers of granulosa cells (corona radiata).

The ovulated oocyte, which enters the fallopian tube, is now arrested at metaphase of meiosis II to prevent parthenogenesis (self-fertilization). For transition of the unfertilized egg to a developing embryo, egg activation is initiated by alterations in Ca${}_{\text{i}}^{2+}$, which is triggered by fusion of the sperm with the egg involving a sperm-derived factor. The type of Ca^2+^-signal is species dependent, a prolonged series of Ca${}_{\text{i}}^{2+}$ oscillations in the egg cytoplasm is characteristic in e.g., mammals, while other species (e.g., fish) exhibit a single increase in Ca${}_{\text{i}}^{2+}$ concentration (Kornbluth and Fissore, 2015). Egg activation comprises a series of events including cortical granule exocytosis, modifications of the zona pellucida and plasma membrane to prevent polyspermy, completion of meiosis II in the egg, recruitment of maternal mRNAs into polysomes for translation, and formation of male and female pronuclei (Schultz and Kopf, 1995; Wang and Machaty, 2013; Swann and Lai, 2016).

### CaSR in ovarian surface epithelial cells

Expression of CaSR in the ovary was first described in human surface epithelial cells (Figure 2A and Table 1). Proteins detected by western blotting show a molecular weight of 120–140 kDa (McNeil et al., 1998). Increasing Ca${}_{\text{o}}^{2+}$ concentrations induced a significant proliferative response in these cells, indicating that the cells can sense Ca^2+^ concentrations in the surroundings. Induced proliferation of these cells due to high Ca^2+^ concentrations may be of physiological relevance during the healing of the ruptured surface following each ovulation, when neighboring cells are locally exposed to ovarian follicular fluid. At least in pig ovarian follicular fluid, Ca^2+^ concentrations of around 2.34 mmol/L have been measured (Schuetz and Anisowicz, 1974); CaSR is active above threshold Ca${}_{\text{o}}^{2+}$ levels between 0.5 and 2 mmol/L (Conigrave and Ward, 2013). Upon treatment of human surface epithelial cells with the CaSR agonists Gd^3+^, Ca^2+^ as well as neomycin, intracellular Ca^2+^ release occurred. The same observation was made in rat ovarian surface epithelial (ROSE) cells. There, inositol triphosphate production increased after stimulation with Gd^3+^ and Ca^2+^. Expression of an interfering mutant CaSR inhibited the proliferative response to elevated extracellular Ca^2+^. CaSR agonists induced tyrosine phosphorylation, ERK activation and proliferation. Expression of interfering mutants for Ras, Raf, and MKK1 indicated that proliferation of ROSE cells in response to increased Ca${}_{\text{o}}^{2+}$involves cross-talk between CaSR and a tyrosine kinase-dependent Ras-Raf-MKK1-ERK signaling pathway. Interestingly, agonists of CaSR also increased the kinase activity of Src, which is a proto-oncogene (Hobson et al., 2000). This work was later extended by Bilderback et al. (2002), who demonstrated an additional ERK-independent, but PI-3K/AKT-dependent component in the proliferative response of ROSE cells. Activation of these pathways has also been observed upon stimulation of CaSR in prostate and Leydig cell lines (see Section CaSR and Disorders of the Male Reproductive System)

Ovarian surface epithelial cells are the cell type primarily responsible for malignant ovarian carcinoma, which is the most lethal female genital malignancy. McNeil et al. (1998) observed increased expression of CaSR mRNA in two ovarian tumor cell lines (BG-1 and CAOV-3). More evidence for a link between CaSR and ovarian cancer is given by the observation that the G allele of the CaSR rs17251221 polymorphism seems to protect against ovarian cancer (Yan et al., 2015). CaSR is considered as a molecule that can either promote or prevent tumor growth depending on the type of cancer (Tennakoon et al., 2016). The exact role of CaSR the development of ovarian cancer remains to be determined.

### CaSR in follicular cells

CaSR is present at the surface of human oocytes and granulosa cells within the corona radiata (Figure 2B and Table 1). Western-blot analysis revealed a single 130 kDa protein in denuded oocytes and a protein doublet of 130/120 kDa in cumulus cells (Dell'Aquila et al., 2006). The expression and localization of CaSR protein in human oocytes at different maturation stages (GV, MI, and MII) was studied by immunofluorescence microscopy. Increased CaSR protein expression in the MI stage as compared to GV and MII oocytes (Figure 2B) suggested a role of CaSR in the process of meiotic maturation, which would correlate with the observed role of external Ca^2+^ in mobilization of intracellular Ca^2+^ during oocyte maturation, activation, and fertilization (review by Tosti, 2006).

CaSR mRNA and protein expression was also shown in equine follicles (Table 1). Again, western blot analysis revealed a single 130 kDa protein in denuded oocytes and a protein doublet of 130/120 kDa in cumulus cells (De Santis et al., 2009). When studied by confocal microscopy, CaSR was demonstrated at the plasma membrane and, more pronounced, within the cytoplasm of oocytes at all examined stages of meiosis (GV, MI, MII). Corona radiata cells exhibited strong plasma membrane labeling. CaSR was also detected in the transzonal cytoplasmic processes of corona radiata cells, which penetrate through the zona pellucida and contact the oocyte membrane. A role of CaSR in oocyte maturation was investigated. The CaSR agonist NPS R-467, which is an allosteric modulator of CaSR sensitizing the CaSR to Ca^2+^ without activating it, in the absence of Ca^2+^, had a stimulatory effect on oocyte maturation. Pre-incubation with the CaSR antagonist NPS 2390 attenuated the effect. Stimulation of maturation by CaSR agonist depended on the presence of external Ca^2+^(2.92 mM) and was not observed at suboptimal external Ca^2+^(0.5 mM). However, variations of Ca^2+^ between 0.5 and 4 mM Ca^2+^ in the absence of the agonist did not stimulate *in vitro* oocyte maturation. In oocytes treated with NPS R-467, CaSR immunostaining increased at the plasma membrane, while it was reduced in the cytosol. Finally, treatment of oocytes as well as cumulus cells with CaSR agonist resulted in an increased activity (phosphorylation) of MAPK (ERK) in these cells (De Santis et al., 2009). Activation of MAPK is necessary for gonadotropin-induced meiotic resumption of oocytes (Fan and Sun, 2004).

CaSR mRNA and protein is also present in porcine oocytes and cumulus cells (Table 1). In contrast to human and equine oocytes, a 160 kD protein was detected by western blotting. The effects of gonadotropins and EGF, two key factors involved in oocytes maturation (Conti et al., 2006; Uhm et al., 2010), on CaSR expression and localization in porcine oocytes were tested. CaSR expression was up-regulated in oocytes matured in gonadotropin-containing, but not EGF-containing medium. Cortical distribution of CaSR was enhanced with gonadotropins but not EGF. Porcine cumulus-oocyte-complexes exposed to CaSR agonist NPS R-568 in gonadotropin-containing medium showed increased maturation rate, while CaSR antagonist NPS 2390 to medium supplemented with gonadotropins significantly decreased oocyte maturation. MAPK (ERK) phosphorylation was increased during *in vitro* maturation as well as after NPS R-568 treatment. Treatment with NPS 2390 resulted in reduced levels of phosphorylated MAPK during oocyte maturation. It was concluded that CaSR participates in gonadotropin-induced oocyte nuclear maturation through MAPK signal transduction (Liu et al., 2015).

Apoptosis of granulosa cells is an important process in follicular atresia. The causal relationship between Ca^2+^ and induction of apoptosis was investigated in cultured avian granulosa cell explants of Japanese quail (*Coturnix japonica*). Increasing extracellular Ca^2+^ resulted in a biphasic response of the cells; an initial inhibitory effect on apoptosis was followed by a delayed phase of increased apoptosis. As the initial inhibitory effect of the Ca^2+^ on apoptosis was mimicked by applying the CaSR agonists Mg^2+^ and Gd^3+^, an involvement of CaSR in inhibition of apoptosis was suggested (Mussche et al., 2000). In a follow up paper, the same group by immunocytochemistry confirmed expression of CaSR in granulosa cells of quail pre-ovulatory follicles as well as in the remnants of the granulosa layer after ovulation (Table 1). CaSR was not detected in the granulosa cells of smaller undifferentiated follicles. The presence of CaSR in follicles destined to ovulate was confirmed by western blotting showing a protein of 115-125 kDa. The rate of apoptosis of F1 granulosa explants (F1 being the largest preovulatory follicle) stimulated by either gonadotropin withdrawal alone or in combination with C_8_-ceramide was significantly decreased with a CaSR agonist (NPS R-568) as well as the ions Ca^2+^ and Mg^2+^. The authors suggested that activated CaSR may play a role in securing the survival of avian follicles after their selection (Diez-Fraile et al., 2010).

### Research demands

Disturbance in oocyte meiotic events can lead to subfertility or premature aging by reducing the functional ovarian reserve. Any molecular target that would allow for promoting or reducing oocyte maturation would therefore be of interest. Expression of CaSR in human, equine, and porcine oocyte has been shown. In analogy to sperms, the molecular weight of the detected CaSR protein in oocytes and granulosa cells differs between the species. Reasons for that are given in Section Research Demands (page 7). Current data support a role of CaSR in maturation (completion of first meiosis) of oocytes and a contribution of the MAPK ERK signaling pathway. However, the interplay of CaSR with other molecules that maintain oocytes in the meiotic arrest and those that initiate meiotic resumption (such as LH) (Celik et al., 2015) is currently not well explored.

The oocyte–somatic cells interaction is very important. Low quality of somatic cells and difficulties in the interaction between oocyte and granulosa cells can reduce the fertilization and implantation capacity of the oocyte/zygote, which again results in poor pregnancy outcome. The expression of CaSR in granulosa cells of various species was confirmed, but current data addressing the function of CaSR in these cells are still limited and point toward an anti-apoptotic function. Expression of CaSR in theca cells remains unexplored. PCOS is a common multifaceted metabolic disease in women of fertile age, which has a strong genetic component. Recent results provided evidence that CaSR Hin1I gene polymorphism represents a candidate for the genetic contribution to the development or the severity of insulin secretion in women with PCOS; So far, the exact molecular mechanism underlying this association is largely undetermined (Ranjzad et al., 2011). It remains to be demonstrated whether theca cells are also affected.

Finally, CaSR expression was observed in epithelial surface cells of ovaries, where it promotes a proliferative response. Up-regulation of the response in case of tumors need to be further explored before therapeutic use of antagonist can be considered.

## CaSR in the uterus

### Uterus and implantation

During mammalian development, parts of the Müllerian ducts develop into the female uterus. The extent of duct-fusion is species-dependent, resulting in different shapes such as simplex uterus in humans and primates, or duplex uterus in rodents such as rats and mice. Irrespective of the shape, the function of the uterus is to enable implantation of the fertilized egg (zygote), to house the growing conceptus and to expulse the fetus during delivery (Spencer et al., 2012).

The uterus consists of a tripled-layered wall, composed of perimetrium, myometrium and endometrium. The endometrium is a mucosal layer which undergoes marked changes in thickness and structure during the estrous cycle in animals such as rats (Westwood, 2008), but most pronounced changes are seen during the menstrual cycle of humans/primates (Fazleabas and Strakova, 2002). The early embryo enters the uterus in the morula state (12–16 cells). Then, transformation into the blastocyst occurs, which is comprised of a layer of trophectoderm cells that contacts the uterine epithelium and starts to form the placenta, and the inner cell mass that gives rise to the embryo. The blastocyst can implant after shedding the zona pellucida; in human, implantation occurs 9 days post coitum (p.c.), while in rats and mice it occurs 4–5 days p.c. The principle purpose of implantation is to enable a contact between maternal blood supply and the developing embryonic blood vessels. The mechanism of implantation of the conceptus is, however, species dependent. Based on the interaction between blastocyst and uterine cells it is classified as being interstitial (e.g., human, guinea pig), centric (e.g., rabbits, domestic animals), and eccentric (e.g., rats, mice) (Lee and DeMayo, 2004). During interstitial implantation the conceptus breaks through the surface epithel of the maternal uterus and invades the underlying stroma. Centric implantation means fusion of blastocyst with uterine epithelium without penetration and in eccentric implantation, the luminal epithelium invaginates and surrounds the blastocyst. Due to implantation, endometrial stromal cells undergo the decidual reaction and become decidual cells (Gellersen et al., 2007).

Successful implantation requires a delicate interplay of many molecules in a limited period of time (the window of implantation). If communication between the embryo and the endometrium fails, then implantation fails. This is an important cause of infertility. There are estimations that only 50–60% of all conceptions advance beyond 20 weeks of gestation and from the pregnancies that are lost, 75% represent a failure of implantation. Failed implantation is also a major limiting factor in assisted reproduction. To treat disorders such as infertility, which increases worldwide due to an increasing toxic environment, but also diseases such as obesity, it is important to understand the molecular mechanisms of implantation and placentation (Norwitz et al., 2001). Many factors including steroid hormones, cytokines, and growth factors, but also Ca${}_{\text{i}}^{2+}$ play a role in the course of implantation (Ruan et al., 2014). Ca${}_{\text{i}}^{2+}$ seems to promote the blastocyste-endometrium interaction (Thie and Denker, 2002). Of interest, the Ca${}_{\text{o}}^{2+}$ concentration in rat uterine secretion changes at the time of implantation (Nilsson and Ljung, 1985).

### Expression of CaSR in the uterus

Xiao et al. (2005) found CaSR mRNA expression in rat uterus (Figure 2A and Table 1). CaSR mRNA and protein appeared in the luminal epithelium on day 1 of pregnancy. Expression of CaSR was switched from the luminal epithelium to stromal cells on days 1–3 of pregnancy. Expression diminished on day 4, but was again induced by the implanting blastocyst. The results suggested that CaSR expression in the stromal cells in the receptive status of the uterus was induced by the implanting blastocysts, while in epithelial cells during day 1 through day 5, the expression of CaSR was regulated by some non-embryonic factors. An embryo transplantation model confirmed that CaSR expression in the uterus was induced by the implanting blastocysts. Artificial decidualization caused upregulation of CaSR expression in the decidualized cells. Furthermore, estrogen as well as progesterone induced the expression of CaSR protein in the uterus. The strong CaSR expression at the implantation site and decidual cells in rat uterus suggests that CaSR is of relevance for blastocyst implantation and decidualization (Xiao et al., 2005).

In addition to the endometrium, also the myometrium, the thick muscular coat of the uterus, has important functions during reproduction as it contributes to sperm and embryo transport, but also to implantation. Ca^2+^ plays an important role for the uterine contractility (Aguilar and Mitchell, 2010). Based on the observation that uterine contractions can be inhibited by the CaSR ligands spermine and high concentrations of Mg^2+^, it was speculated that CaSR was involved in the regulation of myometrial contractility (Pistilli et al., 2012). CaSR expression was confirmed in the estrogen-dominated rat uterus. Highest expression of CaSR was seen in the luminal epithelium, but it was also detected in longitudinal and circular muscle layers of the myometrium (Figure 2A and Table 1). Oxytocin-induced contraction of the rat uterus was not only inhibited by various CaSR agonists such as polyvalent cations and polyamines, but also by two synthetic positive allosteric CaSR modulators, (R)-calindol and (R)-cinacalcet. However, the positive allosteric modulation of CaSR did not show the appropriate stereoselectivity. (S)-cinacalcet, which is usually less potent than the (R)-enantiomer, inhibited contraction to the same extent. Furthermore, the negative allosteric modulator calhex 231 also caused concentration-dependent relaxation. Thus, the pharmacological profile of inhibition of contractility by CaSR ligands was not consistent with their effects being mediated through CaSR, but was rather consistent with promiscuous actions of the ligands. Later, the same authors investigated CaSR expression and function in the pregnant human myometrium. Despite bright staining in the human placenta, only sparse expression of CaSR receptors in pregnant human myometrium was observed (Figure 2A and Table 1). Exposure of human myometrial strips to CaSR receptor ligands showed that calindol and cinacalcet, CaSR agonists, were ineffective as inhibitors of contractions, while the CaSR antagonist calhex 231 produced partial inhibition of contractility (Crankshaw et al., 2013). In summary, the role of CaSR in the rat myometrium remains unclear.

### Research demand

In assisted reproduction, implantation failure is the pregnancy rate-limiting step. The mechanisms underlying human embryo-endometrium signaling are not fully understood, but this is required to improve assisted reproduction outcomes. A detailed understanding of the implantation process is also crucial to develop effective interventions to prevent early pregnancy loss. The small number of studies currently addressing CaSR expression in the uterus demonstrated expression in rat endometrium, but more functional studies confirming the suggested role in implantation and decidualization are required, not only for the rat system, but also for humans and all other species, where assisted reproduction could be of interest. It is known that studies done in animal models do not always translate well to humans. Human *in-vitro* models of implantation (Weimar et al., 2013) may provide an alternative possibility to study human CaSR function during implantation and decidualization. Expression of CaSR in the myometrium was so far only investigated in rat and human. The function of CaSR in the myometrium remains unclear.

## Placenta

### Placenta formation and function

Placentas are unique and transient organs, which develop in female mammals during pregnancy at the interface of maternal and fetal circulation. The organ exhibits an amazing range of morphological variations across species (Benirschke et al., 2012).

Formation of a placenta is of great importance for the development of the embryo and starts very early in pregnancy. The first differentiation process gives rise to trophoblast cells. Trophoblasts initiate implantation of the embryo into the maternal endometrium by interaction with decidual cells of the maternal uterus. By proliferation, invasion and differentiation, trophoblast cells are the most important builders of the placenta. With progression of (human) placenta formation, two distinct populations of trophoblast cells, the villous and the extravillous trophoblast, which exhibit distinct functions, become evident. The expanding chorionic villi of the placenta, which mediate materno-fetal transport of nutrients are covered by a multinucleated layer of syncytiotrophoblast (STB). This cell layer is maintained by proliferation and fusion of the underlying villous cytotrophoblasts. Extravillous trophoblasts, in contrast, invade the uterus and interact with maternal cells. These cells are important for proper placentation.

The process of implantation of the conceptus is species dependent and can be either **invasive** as seen in humans, most primates, dog, cat mouse, rat, or rabbit meaning that the conceptus will break through the surface epithel of the maternal uterus and invades the underlying stroma or can be **non-invasive** (pig, sheep, cow, horse), integrating the uterine epithelium in the placenta. The depths of invasion as well as the degree of proximity between maternal and fetal circulation can vary largely among species. In humans and guinea pigs, the conceptus invades the stroma so deeply that the uterine surface epithelium is restored over it (interstitial implantation). Other species (dog, cat, rat), may invade the stroma only partially and project into the uterine lumen (eccentric implantation). This can result in a contact to other sites of the uterine lumen and additional placental development (e.g., bidiscoid placenta in rhesus monkey, zonary placenta in dog and cat). While in some species the maternal tissue remains relatively intact (dog, cat), meaning that trophoblast cells contact maternal capillary endothelium, in other species, trophoblast cells also invade the maternal endothelium and ultimately bath in maternal blood (human, rabbit, rat, mouse). These types of placentas are classified as haemochorial, which means that all maternal tissue layers are removed and the chorion (i.e., the trophoblast) bathes directly in maternal blood. But even structures of murine and human placentas differ to some extent. Whereas, the term human placenta is monochorial, which means that one continuous trophoblast cell layer, the STB, separates maternal and fetal blood circulation, the murine placenta is trichorial. Moreover, in rodent placenta, but not in human, other primate, or ruminant placentas the primitive yolk sac, which in all species participates in nutrient exchange between the fetal and maternal circulations before the formation of the placenta, is incorporated into the placenta and turns into the intraplacental yolk sac (IPYS). The IPYS is positioned between fetal vessels and maternal blood spaces, well situated for exchange of substances between mother and fetus. It is a bilayered membrane, where smaller parietal or cuboidal cells on a thick basement membrane (Reichert's membrane) overlie maternal blood spaces and vessels, while tall columnar cells on the visceral or endothelial side overly the fetal vessels. Between these layers is the sinus of Duval, which communicates with the yolk sac cavity and the uterine lumen (Metz et al., 1976).

To ensure optimal fetal development, the placenta fulfills a plethora of functions including gas exchange, nutrient transfer, hormone secretion, and immunological functions. Materno-fetal transfer of the ion Ca^2+^ is indispensable for proper development including mineralization of fetal skeleton. About 30 g Ca^2+^ are actively transported across the human placenta, predominantly during the last trimester of pregnancy. As fetal serum Ca^2+^ concentrations are set at significant higher concentrations than maternal serum concentrations, trans-placental Ca^2+^ transport occurs against an extracellular Ca^2+^-concentration gradient. Placental transfer of Ca^2+^ involves various proteins, including Ca^2+^ channels, Ca^2+^-ATPases and intracellular Ca^2+^-binding proteins (Calbindin-D9k). Current knowledge on placental Ca^2+^ transfer as well as regulation of maternal and fetal Ca^2+^ homeostasis have been reviewed in several recent publications (Baczyk et al., 2011; Olausson et al., 2012; Kovacs, 2014, 2015, 2016).

Despite this active Ca^2+^ transfer, the placental trophoblasts still uses Ca^2+^ as a second messenger to activate diverse cellular functions such as differentiation or proliferation, or—in the case of extravillous trophoblasts—also invasion (Baczyk et al., 2011).

### CaSR expression and function in trophoblast and yolk sac cells

An involvement of CaSR in regulation of murine placental Ca^2+^ transfer was confirmed by heterozygous or homozygous ablation of the CaSR gene in mice. *Casr* knock down reduced placental Ca^2+^ transfer. CaSR disruption may, directly or indirectly, downregulate PTHrP or influence the PTHrP effect on the placenta (Kovacs et al., 1998). PTHrP reaches the maternal circulation during pregnancy most likely from the placenta and breasts, as well as possibly from the uterus and fetal tissues. PTHrP significantly stimulates the placental Ca^2+^ transport via proteins involved in transport (Kovacs et al., 1996; Strid et al., 2002; Bond et al., 2008). A Nuf mice with an activating mutation of CaSR exists (Hough et al., 2004), but, placental Ca^2+^ transport has not been analyzed in this strain. In murine placentas, CaSR mRNA was detected by *in situ* hybridization in both types of IPYS cells, but not in the trophoblasts. By immunohistochemistry, using a monoclonal antibody directed against a region of the CaSR that has been deleted in the *Casr*-null mice, the CaSR was found to be expressed in both layers of the IPYS (Table 1). CaSR was also present in the surrounding labyrinth trophoblasts of wt placenta, but was absent in placentas obtained from *Casr*-null mice. Other molecules important for materno-fetal Ca^2+^-transfer, including PTHrP, PTH/PTHrP receptor, calbindin-D9k, or Ca^2+^-ATPase, were also higher expressed in the IPYS of the murine placenta compared to the trophoblasts. Overall, this suggests that IPYS is the important route of Ca^2+^exchange between mother and fetus in the mouse (Kovacs et al., 2002).

**Figure 3:**
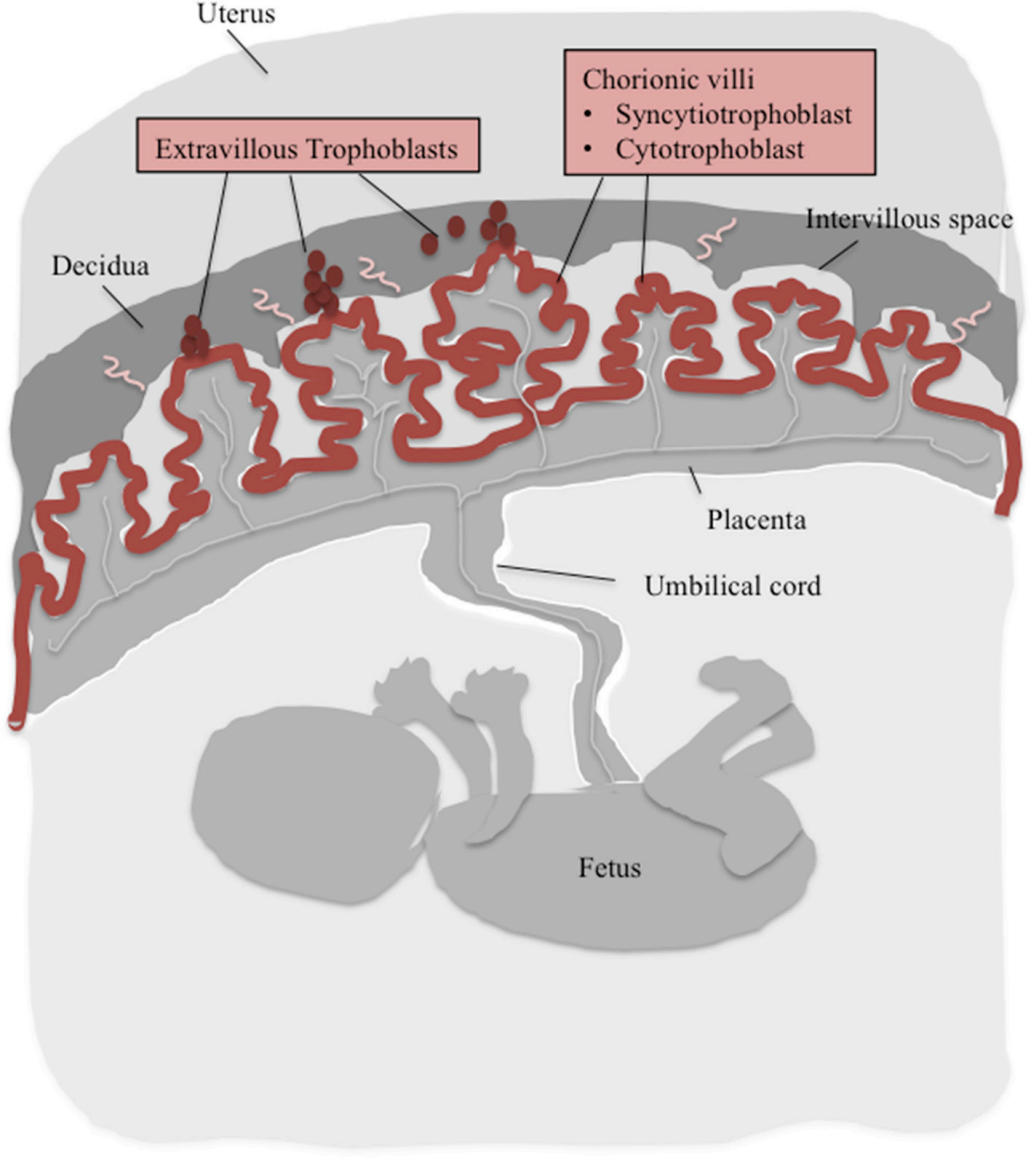
**The cartoon depicts major components of the human placenta**. Cells, which were shown to express CaSR are depicted in red (for details on assumed function of CaSR see Table 1).

Some evidence for involvement of human CaSR in transplacental Ca^2+^-transfer has been obtained recently. Reduced expression of CaSR in placentas derived from women suffering from gestational diabetes mellitus compared with healthy placentas was found. This was associated with lower Ca^2+^ levels measured in cord blood of infants from women suffering from gestational diabetes mellitus supporting its role in placental Ca^2+^-transfer (Papadopoulou et al., 2014). In humans, expression of CaSR was found in both villous and extravillous tissue of first trimester and term placentas (Table 1). In chorionic villi, CaSR was mainly detected at the apical membrane of the STB, which contacts maternal blood and at lower levels in cytotrophoblast cells (Figure 3). This location would be in line with the control of placental Ca^2+^movement. The CaSR was also localized in extravillous trophoblast cells in close proximity to maternal blood vessels (Figure 3). It was speculated that CaSR senses maternal extracellular Ca^2+^ levels during the process of placentation and participates in regulation of placental development (Bradbury et al., 2002). Earlier, Bradbury and coworker had demonstrated that isolated human extravillous cytotrophoblasts expressed the full-length transcript of CaSR. A splice variant lacking exon 3 was also found, which encoded a truncated protein of 153 amino acids (compared with 1078 amino acids for the full length protein). Upon translation, this CaSR splice variant, however, would not be incorporated into the plasma membrane. The extravillous cytotrophoblasts responded to elevation of extracellular Ca^2+^, and also to extracellular Mg^2+^ with a bi-phasic elevation of intracellular Ca^2+^ (Bradbury et al., 1998).

### Research demand

CaSR modulates transplacental Ca^2+^ transfer, at least in the mouse. Some evidence also exists for a role in human transplacental Ca^2+^ transfer. In breast cells, CaSR is expressed in the basolateral membrane of the lactating alveolus and regulates PTHrP secretion. CaSR senses the availability of Ca^2+^ for milk production, and stimulates production of PTHrP when the Ca^2+^supply is insufficient. CaSR also impacts on a Ca^2+^-ATPase (PMCA2) to stimulate Ca^2+^ secretion into milk when the Ca^2+^ supply is adequate (Kovacs, 2016). In the murine placenta, CaSR impacts on PTHrP, but detailed information of CaSR function in the murine or human hemochorial placentas is lacking. Information on expression of CaSR in the epitheliochorial placentas of other species is lacking.

CaSR can regulate proliferation, differentiation or apoptosis, but neither in extravillous nor villous human trophoblasts this issue has been addressed so far. Expression of CaSR in extravillous trophoblasts suggests an important role in placentation. In this context, not only Ca^2+^, but also other CaSR ligands such as L-amino acids (Conigrave et al., 2000) may be modulators of placental tissue development in response to e.g., maternal Ca^2+^ concentration or maternal diet. Signaling pathways targeted by CaSR in the placenta are also unknown. A detailed understanding of the modulators of placentation is necessary before effective interventions can be considered.

Finally, it remains to be demonstrated whether CaSR is the only relevant Ca^2+^-sensing protein of the placenta. In 1989, Juhlin and colaborators generated several monoclonal anti-parathyroid antibodies (E11, G11, B6) by immunization of mice with intact parathyroid cells. Two of these antibodies (G11 and B6) interfered with the Ca^2+^-sensing mechanism of parathyroid cells (Juhlin et al., 1987). When monoclonal antibody E11 was applied in immunohistochemistry on placenta and uterus of the pregnant rat, positively stained rat placental cells were found at the end of pregnancy. Staining was confined exclusively to the columnar epithelial cells lining the sinuses of Duval, i.e., in the IPYS. In the uterus, positive staining of the epithelium lining the uterine lumen was obtained prior to and during implantation (days 5–6) (Bernadotte et al., 1989). When G11 and E11 were applied on sections of human placenta, both antibodies labeled the cytotrophoblast cells of anchoring and floating villi as well as cytotrophoblasts in the chorionic plate. The STB, however, was not stained. Isolated cytotrophoblast were found to react with G11 and E11. In these cells, a temporary increase of Ca${}_{\text{i}}^{2+}$ upon elevation of external Mg^2+^ was observed, which was blocked by G11 antibodies. Raised extracellular Ca^2+^ inhibited release of PTHrp from the cells, and this inhibition was blocked by the G11 antibody (Hellman et al., 1992). E11 was also used in immunoelectron microscopical studies showing positive staining of cytotrophoblast cells of the human placenta, and trophoblast cells lining fetal blood vessels in the rat placenta (Bjerneroth et al., 1992). Both antibodies, however, detected a 500 kDa protein, which is much larger than CaSR (Juhlin et al., 1990). When the protein was then cloned from human placenta, it was found to belong to the LDL-receptor superfamily of glycoprotein and it was identified as gp330 (megalin, LRP2) (Lundgren et al., 1994; Hjälm et al., 1996). In addition to expression in human parathyroid cells, kidney proximal tubule cells and placental cytotrophoblasts, the protein was also detected in other reproductive tissues such as epididymal epithelial cells and mammary epithelium. It was suggested to have Ca^2+^ sensing functions (Lundgren et al., 1997). Unfortunately, further investigations to clarify the role of this protein in Ca^2+^ sensing were not performed.

## Summary and outlook

CaSR is expressed in many cells of male and female reproductive organs. Due to its functional diversity, CaSR could be involved in a variety of reproductive processes ranging from proliferation or maturation of germ cells to implantation of the zygote, and from placentation to transplacental transport processes. Apart from physiologic actions, current data also suggest a role of CaSR in diseases of reproductive organs or pregnancy. Currently, however, we know very little about CaSRs physiologic and pathophysiologic functions in reproduction. Exploration of CaSR function in the context of diseases of reproduction such as male and female infertility and early pregnancy loss, PCOS, or gestational diabetes mellitus as well as tumors of reproductive organs may add novel possibilities for diagnosis and treatment.

## Author contributions

IE designed and wrote the article and meets all criteria for authorship.

## Funding

The author is funded by the EU Marie Sklodowska Curie Action grant CaSR Biomedicine (675228).

### Conflict of interest statement

The author declares that the research was conducted in the absence of any commercial or financial relationships that could be construed as a potential conflict of interest.

## Footnotes

^1^http://www.who.int/mediacentre/factsheets/fs312/en/ (Accessed June 04, 2016).

## Abbreviations

AC: adenylyl cyclase
cAMP: cyclic adenosine monophosphate
EGFR: epidermal growth factor receptor
Gd^3+^: Gadolinium
GV: germinal vesicle
LH: luteinizing hormone
MAPK: mitogen-activated protein kinase
Mg^2+^: Magnesium
PI-3K: phosphoinositide 3-kinase
PLC: phospholipase C
PKC: protein kinase C
CaSR: Calcium-sensing receptor
Ca^2+^: Calcium
IPYS: Intraplacental yolk sac
ROSE cells: Rat ovarian surface epithelial cells
STB: Syncytiotrophoblast
PCOS: polycystic ovarian syndrome
PTH: Parathyroid hormone
PTHrP: Parathyroid hormone-related protein
PY: tyrosine phosphorylation.
